# Ranked retrieval of segmented nuclei for objective assessment of cancer gene repositioning

**DOI:** 10.1186/1471-2105-13-232

**Published:** 2012-09-12

**Authors:** William J Cukierski, Kaustav Nandy, Prabhakar Gudla, Karen J Meaburn, Tom Misteli, David J Foran, Stephen J Lockett

**Affiliations:** 1 Rutgers University, New Brunswick, NJ, 08903, USA; 2 Cancer Institute of New Jersey, New Brunswick, NJ, 08903, USA; 3 University of Medicine and Dentistry of New Jersey, New BrunswickNJ, 08903, USA; 4 Optical Microscopy and Analysis Laboratory, Advanced Technology Program, Science Applications International Corporation-Frederick, Inc., Frederick National Laboratory for Cancer Research, Frederick, MD, 21702, USA; 5 National Cancer Institute, National Institutes of Health, Bethesda, MD, 20892, USA

## Abstract

**Background:**

Correct segmentation is critical to many applications within automated microscopy image analysis. Despite the availability of advanced segmentation algorithms, variations in cell morphology, sample preparation, and acquisition settings often lead to segmentation errors. This manuscript introduces a ranked-retrieval approach using logistic regression to automate selection of accurately segmented nuclei from a set of candidate segmentations. The methodology is validated on an application of spatial gene repositioning in breast cancer cell nuclei. Gene repositioning is analyzed in patient tissue sections by labeling sequences with fluorescence *in situ* hybridization (FISH), followed by measurement of the relative position of each gene from the nuclear center to the nuclear periphery. This technique requires hundreds of well-segmented nuclei per sample to achieve statistical significance. Although the tissue samples in this study contain a surplus of available nuclei, automatic identification of the well-segmented subset remains a challenging task.

**Results:**

Logistic regression was applied to features extracted from candidate segmented nuclei, including nuclear shape, texture, context, and gene copy number, in order to rank objects according to the likelihood of being an accurately segmented nucleus. The method was demonstrated on a tissue microarray dataset of 43 breast cancer patients, comprising approximately 40,000 imaged nuclei in which the *HES5* and *FRA2* genes were labeled with FISH probes. Three trained reviewers independently classified nuclei into three classes of segmentation accuracy. In man vs. machine studies, the automated method outperformed the inter-observer agreement between reviewers, as measured by area under the receiver operating characteristic (ROC) curve. Robustness of gene position measurements to boundary inaccuracies was demonstrated by comparing 1086 manually and automatically segmented nuclei. Pearson correlation coefficients between the gene position measurements were above 0.9 (*p *< 0.05). A preliminary experiment was conducted to validate the ranked retrieval in a test to detect cancer. Independent manual measurement of gene positions agreed with automatic results in 21 out of 26 statistical comparisons against a pooled normal (benign) gene position distribution.

**Conclusions:**

Accurate segmentation is necessary to automate quantitative image analysis for applications such as gene repositioning. However, due to heterogeneity within images and across different applications, no segmentation algorithm provides a satisfactory solution. Automated assessment of segmentations by ranked retrieval is capable of reducing or even eliminating the need to select segmented objects by hand and represents a significant improvement over binary classification. The method can be extended to other high-throughput applications requiring accurate detection of cells or nuclei across a range of biomedical applications.

## Background

In this paper, a simple, fast, and highly accurate pattern recognition technique is introduced to perform segmentation assessment for the purpose of subsequently quantifying gene repositioning. The term “segmentation assessment,” as described here, refers to evaluation of segmentations at the object level, as opposed to the more commonly encountered task of segmentation evaluation, where the goal is characterization of the pixel-wise boundary accuracy [1]. Assessment is an important step whenever accurate segmentation is critical to quantitative, downstream analysis. Whether it is spatial analysis of gene location, counting cells, or performing computer-aided diagnosis on tissue samples, better starting segmentations eliminate errors later in the analysis.

In order to understand the need for segmentation assessment, it is helpful to understand its motivating applications. There are many high-throughput/high-content applications which depend on proper segmentation. Often, these are problems for which there are more imaged cells than necessary for quantitative evaluation of the tissue. The gene repositioning application presented in this manuscript is only one example; other applications include HER2/neu expression in breast cancer prognosis [2], gamma-H2AX measurement for radiation exposure [3], and DNA content analysis [4]. While this work demonstrates segmentation assessment for the purpose of gene repositioning, the general approach is applicable to problems where ranking by segmentation quality is advantageous to subsequent measurements.

### Gene repositioning

Nuclear compartmentalization and the position of specific genes in the nucleus have been shown to impact gene expression and cell function [5]. Nuclear organization is thought to affect cellular activities such as replication, repair, transcription, and breakage-rejoining events [6,7]. Spatial arrangements of chromatin in the nucleus play a critical role in transcriptional regulation [8-11]. Similarly, chromosome territories and chromosomal sub-regions have been shown to have non-random radial nuclear distributions [12-15].

Recent studies focusing on the organization of individual genes have illustrated the tendency of genes to occupy specific spatial positions. Their transcriptional activity sometimes correlates with location in the nucleus and proximity to other nuclear bodies [16-21]. There is evidence that spatial organization of individual genes can potentially be used for detection of breast cancer [22-24], though further work is necessary to determine the suitability of gene repositioning to clinical applications. High-throughput screening, which aims for automation without sacrificing accuracy, will play a key role in this research [25].

Current techniques used for studying the gene position effect in cells involve analyzing multi-color fluorescence images of DNA sequences of genes of interest, labeled by FISH [7,26-28]. Because of natural variability in gene positioning in nuclei, small positional changes cannot be visually observed in individual nuclei. However, the change may be observed as an ensemble phenomenon when quantified across many nuclei per sample. Automatic quantification requires accurate nuclear segmentation, identification of FISH signals, and localization relative to the nuclear boundary. Once the FISH signals are localized, statistical hypothesis tests determine whether the radial distance distribution of a test sample is different than that of a control sample.

Since the radial position is a statistical quantity influenced by several sources of error, the gene localization process mandates a large number of nuclei. Tissue sectioning creates one source of error by presenting a random cross section of cell nuclei. The tissue also shears when cut. Even under ideal preparation conditions and perfect segmentation, a considerable amount of tissue will simply be unusable for spatial analysis due to cell clumping, overlap of boundaries, and the presence of artifacts. It is therefore not sufficient to have a strong segmentation method by itself, since even the most reliable approaches result in errors. Hence, there is need for an assessment step to screen out objects that are not true nuclei or have been improperly segmented. If gene repositioning is deployed as a tool in a diagnostic setting, manual quality control may be necessary to ensure correctness. A method which can reduce this burden without sacrificing accuracy will save time and reduce tedium, resulting in fewer mistakes.

### Segmentation assessment

**Figure 1:**
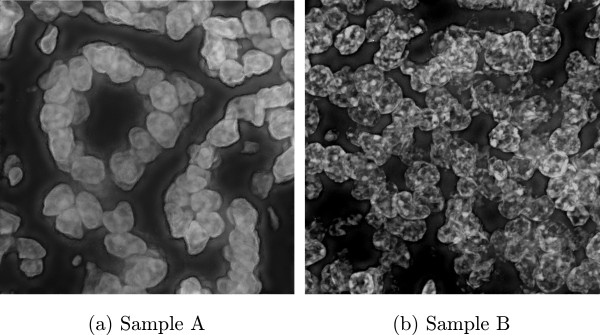
(a,b) There are significant differences in sample appearance, even for tissue processed under identical conditions.

It is reasonable to ask why it is necessary to include a step for segmentation assessment, as opposed to directly improving the segmentation reliability. Why not focus on eliminating errors early in the workflow, so that they do not need to be detected later in the process? Robust image segmentation often falls short of expectations, due to the enormous sample variability encountered in histopathological images. For example, Figure 1 shows images from two different tissue microarray (TMA) cores which have been processed under identical staining and acquisition parameters. Such unpredictable variations in sample appearance may require consensus approaches using different algorithms, adaptive techniques, and different parameters to satisfactorily segment [29-31].

Methods that assess the performance of segmentation methods can partially overcome these limitations. Along these lines, previous work on probabilistic assessment of nuclear segmentation has been performed by Lin et al. [29] and by Gudla et al. [32], albeit for use in the actual segmentation workflow, as opposed to a postliminary feedback step. Lin et al. used a Bayesian method to assign a confidence score for merging multiple segmentation models. Hill et al. [4] showed that a shape-based filter improved the quality of results from high-content screening using the SK-BR-3 cell line, a line which is particularly difficult to automatically segment. Nandy et al. [23] applied a postliminary binary classification via a neural network to separate a subset of well segmented nuclei.

### Ranked retrieval

**Figure 2:**
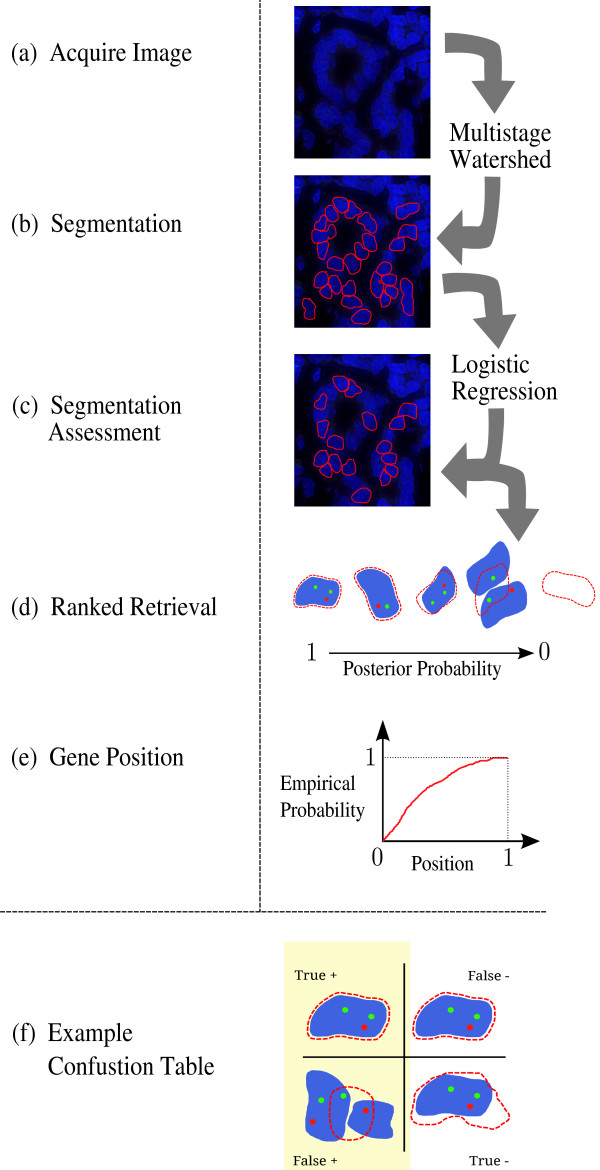
**Overview of the automated steps to extract gene position information from images, which can then be employed for high-throughput studies.(a)** The fluorescence image is acquired. **(b)** A multistage watershed segmentation algorithm creates a set of candidate segmentations. **(c)** A logistic regression assigns a probability to each candidate, screening out those with a low likelihood of being well segmented. **(d)** Examples of highly ranked vs. poorly ranked segmented objects. Blue: true segmentations of nuclei. Red border: automatically deduced candidate segmentations. Green/Red dots: FISH-labeled genes **(e)** Gene position measurements, such as the radial probability distributions, are made using the correctly segmented nuclei borders. **(f)** A confusion matrix illustrating potential outcomes of a binary classification. The red dotted line represents a candidate segmentation. False positives are the most critical source of error in a ranked retrieval of nuclei, potentially creating incorrect gene position measurements.

Segmentation assessment fits into the proposed workflow as follows. After nuclei are segmented, feature extraction is performed on the candidate nuclei and logistic regression used to assign a probability that a candidate segmentation is correct. Nuclei are sorted according to this probability, in order to generate a ranked list. Well segmented nuclei are then used for gene localization analysis, a process described in detail in the Methods section. An illustrated overview of this process is provided in Figure 2.

The identification and selection of a comparatively small number of well-defined/relevant objects from a much larger set of poorly-defined/irrelevant ones is an application which can be reliably addressed by ranked retrieval. Instead of imposing a binary good/bad label on each object, a probability leads to a relative ordering. From this perspective, the goal is not only to minimize the global classification error across the population of nuclei, but also to sort nuclei according to a quality of segmentation. This ranking should be based on a calibrated probability, meaning that it represents the true probability that an object is a well segmented nucleus. The Methods section describes logistic regression and justifies its suitability to high-throughput applications. Ranked retrieval additionally suits a task such as gene localization because, despite the low yield of usable nuclei per image, one can generally compensate by acquiring more images. A single TMA core is generally sufficient to reach the number of usable nuclei necessary for statistical hypothesis testing [23,24].

Given a set of nuclei sorted according to a probability of being well segmented, there are several ways of selecting the nuclei usable for gene localization analysis (e.g. by taking the top *N* nuclei per subject, taking nuclei above a probability threshold, or using a weighted sum). Since there is generally a surplus of nuclei, failure to identify usable nuclei (false negatives) is generally tolerable, provided they are not biased in some way. This leaves false positives as the problematic source of error. This is shown visually in Figure 2c, where it is evident that, for localization analysis, specificity (that the segmented nuclei are correct) takes priority over sensitivity (that all the good nuclei were found). In this study, we demonstrate that the ranked retrieval is both more accurate than binary classification and less burdensome for an expert to review.

## Methods

### Data preparation

A trained expert acquired over 1700 images from a breast cancer TMA (Biomax). 4*μm* thick formalin fixed, paraffin-embedded sections were imaged using an Olympus IX70 microscope using a 60X, 1.4 oil objective lens and an auxiliary magnification of 1.5. 3-D Z-stacks were acquired with a step size of 0.5*μm*. The image size was 1024 x 1024, with a pixel size of 0.074*μm*per pixel in both X and Y directions. Nuclei were stained with 4’,6-diamidino-2-phenylindole (DAPI) and the *HES5* and *FRA2* genes labeled by FISH. For nuclear segmentation, maximum intensity projections of the original DAPI (blue) channel were used, as previously described in [24]. The data comprised a cohort of 43 subjects aged 16 to 68, with 1 patient with hyperplasia, 2 fibroadenomas, 1 invasive papillary carcinoma, and 39 invasive ductal carcinomas. Both node negative and positive tumors were included in the invasive carcinomas, with grades from I to III. Both AR+/-, ER+/-, PR+/-, P53+/-, KI67+/- and HER2+/- tumors were included.

Automated segmentation was performed via a multistage watershed algorithm followed by a tree-based hierarchical merging procedure using shape models [33]. We refer the reader to [23] for a detailed explanation of the segmentation methodology. After segmentation, a total of 43,956 candidate nuclei were analyzed.

### Manual annotation of segmented objects

Elimination of inter and intra-observer variability is a significant benefit of automated image analysis, yet the extent of this variability is seldom quantitatively measured. Human annotation serves two roles in this paper. First, it provides the ground truth for the training and performance characterization of the logistic regression. Second, it estimates the baseline variability associated with choosing properly segmented nuclei.

Humans assigned a label from one of three categories for the segmentation of each nucleus, according to the following criteria: 

**Good: 1** (usable for FISH localization) boundary is almost perfect, not multi-nuclear, relatively small inclusions/extrusions allowed, nucleus is not occluded

**Maybe: 0.5** (possibly usable for FISH localization) boundary has minor errors, not multi-nuclear, nucleus may be partially occluded, clipped, or out of focus

**Reject: 0** (not suitable for FISH localization) boundary is incorrect, may be multi-nuclear, occluded nuclei, nuclear fragments, debris, background

Annotation was performed using a custom graphical user interface, which displayed the mask, best-fit ellipse, contour, and grayscale image of each nucleus, as well its context in the image. The FISH signals of the specific gene were not used in evaluating the segmentation, however they were included as features in the automatic classification. This is because the FISH signals had no influence on the segmentation, but were useful for determining which segmentations are genuine nuclei. For example, an abnormally high number of FISH spots likely indicates that multiple nuclei have been incorrectly segmented as one object.

### Feature extraction

Four categories of features were extracted and tested in the automatic pipeline: morphological (relating to the shape of the segmentation boundary), textural (relating to the intensity inside the nucleus), contextual (relating to the relative layout of the nuclei in an image), and gene-based (relating to the number, distribution, and properties of the labeled genes). The features ranged in complexity from simple geometric and statistical descriptions, to more advanced parameters relying on morphological operations, elliptical Fourier coefficients [34], corner detectors, and fractal dimension. Features are listed and briefly summarized in Table 1. A more detailed explanation of each is given in the Additional file 1.

**Table 1:** Four categories of features were extracted and tested in the automatic pipeline

**Category**	**Name**	**Brief description**
	Area	Number of pixels in segmentation mask
	Perimeter	Length of segmentation boundary
	Perimeter/Area	Perimeter to area ratio
	Solidity	Ratio of area to the convex hull area
	CH Perimeter / Perimeter	Ratio of convex hull perimeter to object perimeter
	Max Circle Ratio	Ratio of the area of the largest inscribed circle to the total area
	Ellipse Eccentricity	Eccentricity of the ellipse that has the same second-moments as the mask
Morphological	Ellipse Error Ratio	Area difference between best-fit ellipse and boundary
	Length	Measure of elongation
	Width	Measure of breadth
	Mean Pairwise Distance	Mean all-pairs distance between points on perimeter
	Polar Histogram	Measures isotropy of border points at a given angle
	Num. Severe Corners	Number of strong corners in segmentation contour
	Box-counting Dimension	Fractal dimension of the perimeter
	Erosion Profile	Identifies segmentations with narrow passages separating large areas
	Elliptical Fourier	Number of elliptical Fourier coefficients to reconstruct the mask to within
		10% area error
	Mean Intensity	Mean of grayscale intensity inside nucleus
Texture	Intensity Range	Range of grayscale intensity inside nucleus
	Entropy	Global entropy of grayscale values inside nucleus
	Gray-level Co-occurrence	Statistics of the gray-level co-occurrence matrix
	Num. FISH	Number of FISH spots
	FISH/Area	Number of FISH spots normalized by area
FISH	FISH CH Area	Ratio of convex hull area formed on FISH spots to total area
	FISH Boundary	Measures whether the FISH convex hull intersects the nuclear boundary
	Mean FISH Distance	Mean distance between FISH spots
	Num. Nuclei	Number of candidate nuclei in the image
	Intensity Ratio	Mean intensity of band surrounding the nucleus compared to the mean intensity inside
Contextual	Betweenness Centrality	Betweenness centrality of nucleus in a graph connecting nuclei in the image
	Number Neighbors	Number of neighbors connected to the nucleus
	Mean Edge Distance	Mean edge distance to 1st-level neighbors

^a^Summarized here are brief descriptions of the features.

A random forest [35] was run to determine the out-of-bag importance of the individual features. In general, morphological features ranked highest, followed by texture-based, then FISH-based, and finally contextual features. The most discriminatory features captured variations on the perimeter-to-area ratio, whether measured directly or through indirect quantities, such as the solidity or fractal dimension. Contextual features were found to be unreliable due to the variation in performance of the segmentation algorithm on datasets of different quality. Due to their poor performance, high cost of computation, and the input/output burden to retrieve full images, contextual features were omitted from subsequent analysis. Feature extraction was implemented in a naively parallel way on a cluster and took on the order of a few seconds per nucleus.

### Logistic regression

After manual annotation of a training set and feature extraction, a supervised machine learning algorithm was introduced to perform the segmentation assessment. Logistic regression is a form of generalized linear model that models the posterior probability of a dichotomous or continuous target variable [36]. To compute this probability, regression is performed against the logarithm of the odds ratio,

(1) $$\ln\left(\hat{p}/(1-\hat{p})\right)=\alpha +\beta_{1}x_{1}+\beta_{2}x_{2}+\ldots +\beta_{m}x_{m},$$

where the *x*_*i*_ are the input features and *β*_*i *_the regression coefficients. Explicitly solving for the probability gives,

(2) $$\hat{p}=\frac{e^{\alpha +\beta_{1}x_{1}+\beta_{2}x_{2}+\ldots +\beta_{m}x_{m}}}{1+e^{\alpha +\beta_{1}x_{1}+\beta_{2}x_{2}+\ldots +\beta_{m}x_{m}}}.$$

Logistic regression has several theoretical and practical properties which suit the task of segmentation assessment for gene position analysis. Chiefly, it allows direct comparison of probabilities across different training sets. This is a desirable property that not all prediction methods share. For example, it has been established that margin-based methods (e.g. boosted trees or support vector machines) and models such as Naive Bayes produce distorted outputs, which require a calibration step to align the ranking with the true class posterior probability [37,38]. Conversely, the logistic regression output aligns with the true class posterior probability and does not necessitate a calibration step [38]. For this application, the probability assigned via regression reflects the real probability that an object is a well segmented nucleus. In terms of implementation, logistic regression is fast, conceptually simple, and scales to large data. One needs only to save the regression coefficients for storage and future use. The target variable can be a dichotomous outcome (e.g., whether to use or ignore a candidate segmentation), or, in the case of multiple ground truths, a probability in [0,1]. Each nucleus is treated as a separate data point in this proposed method, yielding ample training data.

### Ranked retrieval

The logistic regression was trained and applied in a leave-one-out fashion. Each subject’s tissue samples were held out and the regression trained on the remaining subjects. Leave-one-out cross validation was chosen to approximate a clinical scenario, in which a given subject’s gene position measurements would be compared to a group of prior subjects with known outcomes. This process was repeated for each of the 71 TMA cores from the 43 subjects. Due to the difficulty of segmenting the images used for this study, there were many more rejected segmentations in the ground truth (84%) than well-segmented nuclei (16%). The posterior probability ranking from the regression was scored against the averaged ground truth from the three manual annotations.

### Dataset quality

There are three hierarchical levels into which nuclei may be grouped: each nucleus belongs to a patient, a tissue sample from that patient, and an image from that tissue sample. While the distinction of which nuclei fall into which image is at the discretion of the microscopist, the other groupings impose real, practical constraints on the data analysis. For example, the quality and quantity of tissue across different specimens may produce vastly different datasets, one with plentiful, easy-to-segment nuclei and the other with scant, clumping nuclei buried in background fluorescence. Even though the end goal is the same (an adequate number of well-segmented nuclei), there is no fixed number of images necessary to arrive at this end.

**Figure 3:**
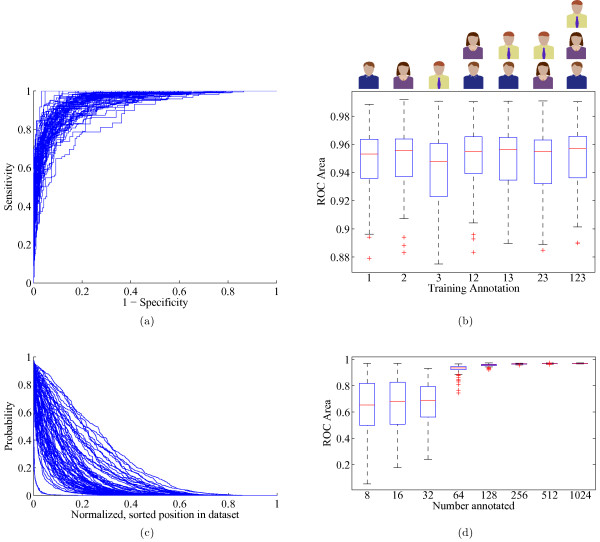
**(a) ROC curves for each leave-one-out experiment trial, with the ground truth taken from all three reviewers (b) ROC performance as a function of the number of reviewers (c) Sorted values of the posterior probability values for each dataset, scaled to the domain [0,1].** The area under these curves is a representation of the yield of well segmented nuclei from the dataset. **(d)** The effect of training set size on the regression performance, reported for 500 repetitions.

The varying yield of good nuclei across different tissue sections is accommodated by introducing a measure of dataset quality using the aggregate posterior probabilities of the logistic regression. The probabilities are sorted and normalized by the number of candidate segmentations in each dataset. The area under this curve yields a number in [0,1], roughly corresponding to the proportion of the dataset assigned a highly correct segmentation probability, $\frac{1}{N}\underset{i\in S}{\sum }{\hat{p}}_{i}$, with $\hat{p}$ the estimated probability, *N* the number of total candidate segmentations, *S*, across all images belonging to the subject. Example quality curves are plotted in Figure 3c. This quality number is proposed to guide the microscopist regarding the usability of the specimen under examination. Datasets which are difficult to segment have a low quality score, signaling a poor success rate of segmentation and the need to acquire more images. Although the concept of quality/yield is a procedural matter affected by numerous, unrelated causes (underlying pathology, histology, sample prep, acquisition settings), it must be accommodated in order to automatically segment nuclei in statistically meaningful numbers.

### Measuring gene centrality

We introduce three successive methods to measure gene centrality, each incorporating a varying degree of global shape information. Let the set *c*_*ij *_∈* C* be the points comprising the segmentation contour and *x*_*ij *_∈* M *the set of points enclosed by the contour (the mask). The euclidean distance is denoted by *d*(·). The nuclei screened by the aforementioned methods were used for gene localization.

#### EDT (local)

The Euclidean distance transform (EDT) [39,40] is defined as the smallest distance from the point of interest to any point on the perimeter,

(3) $$\text{edt}(x_{\text{ij}})=\underset{C}{\min}d(x_{\text{ij}},C).$$

Points on the boundary have a value of zero. Unlike more primitive methods, which employed a radial distance from a central point, the EDT is shape invariant and does not impose a circular/ellipsoidal assumption on the cells. However, to facilitate comparisons across different cells, it is desirable to have a centrality measure which is both scale and shape invariant.

#### Normalized EDT (local/global)

The standard method to achieve scale invariance is by dividing the EDT by the largest EDT value of any point in the mask. This results in a value of zero at the periphery. Although the literature often uses this convention, it is useful to use one minus this quality so that the central points (“the origin”) take a value of zero and the periphery a value of one,

(4) $$\text{nedt}(x_{\text{ij}})=1-\frac{\text{edt}(x_{\text{ij}})}{{\text{max}}_{M,C}d(M,C)}.$$

A similar approach employs an average radial distance for normalization, computing the radius of a circle with equal area to the segmentation [41]. The normalized EDT (nEDT) is partially shape and scale invariant, but is not without drawbacks. The maximum EDT value provides only a rough correction for object size, meaning the normalized EDT is not directly comparable for objects of different shape [27,42]. This can be corrected by expressing the distribution of the interior points as a cumulative frequency.

#### Cumulative EDT (global)

A scale and shape invariant centrality measure is constructed by using the empirical cumulative probability distribution from the nEDT values given by Eqn. 4. This describes the empirical probability that points in the nucleus have a nEDT value less than or equal to a given value,

(5) $$\text{cedt}(x_{\text{ij}})=P\left[\text{nedt}(M)\leq \text{nedt}(x_{\text{ij}})\right].$$

This method was used by Schwarz-Finsterle et al. [28] and later by Andrey et al. [43] to characterize relative distances of FISH distributions within cells. The cumulative EDT (cEDT) does not rely on an *a priori* shape model. Therefore, this approach is appropriate for highly irregular shapes, can easily be extended to three dimensions, and does not require specification of spatial parameters.

## Results

### Manual agreement

Three human reviewers independently annotated the dataset with one of three labels, {good, maybe, reject}, as described in the Methods section. The three labels were averaged and used as a continuous target variable for performing regression. With three label values (0, 0.5, 1) and three annotations (*y*_1_, *y*_2_, *y*_3_), there were five possible aggregate label values: 1, 2/3, 1/2, 1/3, 0.

Significant variation was observed in the labels assigned by the human reviewers. As a more concise measure of agreement, Receiver-Operator characteristic (ROC) curve areas of manual labels were constructed by averaging two as ground truth and scoring the third against those two (Table 2). A detailed confusion matrix is provided in Additional file 1. The mean ROC agreement was approximately 0.9, indicating subjective differences in the opinion of what constitutes a well segmented nucleus, as well as a high level of difficulty in the segmentation task. This value is provided as a baseline ROC area for comparison with the automated ranking on this dataset. Note that agreement was computed in an object-wise fashion, which is related to but not the same as the pixel-wise segmentation error.

**Table 2:** ROC values of manual labels were constructed

**Ground truth**	**Predicted**	**ROC area**
(*y*_1_ + *y*_2_)/2	*y*_3_	0.868
(*y*_2_ + *y*_3_)/2	*y*_1_	0.905
(*y*_1_ + *y*_3_)/2	*y*_2_	0.936

^a^ROC areas for the manual agreement (*N *= 43,956).

### Retrieval results

Retrieval accuracy was quantified by ROC analysis, scoring the posterior probability from each hold-out trial against the ground truth. The mean ROC area performance across all 71 logistic regression trials was 0.95, with a worst case of 0.89 and a best of 0.99 (Figure 3a). The automated approach consistently outperformed the mean human ROC scores. The leave-one-out experiment was run using all permutations of the three sets of manual labels in order to test the effects of using multiple training annotations (Figure 3b). The best performance came from using all three sets, though the performance was only marginally better than using just two, or even a single set of annotations. In particular, the ground truth from reviewer #2 alone scored well in both the mean ROC area, as well as having smaller variance across the different data folds.

Counterintuitively, dataset quality was negatively associated with the ROC area (*r *= − 0.32). Visual inspection confirmed that for the low quality, “messy” images, the segmentation made such egregious mistakes that the pattern recognition could more easily identify well segmented nuclei. These tended to be ellipsoidal-shaped nuclei surrounded by background. The steep decay in the majority of the quality curves (Figure 3c) reflects the difficulty of the automated segmentation task and corresponds to the high percentage of objects rejected by manual annotation.

Both random forests and linear kernel support vector machines (SVM) were tested in addition to logistic regression. The posterior probability from the random forests yielded similar performance to logistic regression, but at the cost of increased runtime. The SVM proved too slow to use for the 71-fold holdout experiment and additionally did not perform as well on smaller subsets of the data (data not shown). For all three methods, using a ranked probability outperformed binary classification, due to the ability to assign an order within each class. We hypothesize the comparable results between methods to be the consequence of an expressive feature space, one which makes the classes separable without the need for kernel tricks or excessively complicated learning strategies. Scatter plots of the data (not shown) indicated clustering of the good/maybe nuclei in feature space.

### Effect of ground truth size on accuracy

Another experiment was performed to determine how many nuclei must be manually annotated in order to reach the asymptotic best performance. Random subsets of sizes ranging from 8 to 1024 nuclei were selected as a training set, with the remaining nuclei used as the test set. Logistic regression was performed and the ROC area recorded. This was repeated 500 times for each subset size, using a new random training set each time. Results showed that as few as 256 training points are necessary to reach near-optimal performance (Figure 3d). Including more than 256 training points reduced the variance introduced by drawing random data subsets, thus making the training set more likely to be a representative sample of the total population.

### Comparison to manual analysis

In order to validate the efficacy of the regression-based nuclear screening method, FISH localization results of the *FRA2* and *HES5* genes for 13 datasets were compared to those obtained by an independent, complete manual analysis (in which cells were selected, segmented, and the FISH spots marked by hand). Because it was infeasible to process the entire TMA by hand, a subset of 13 datasets was chosen. Table 3 indicates the 13 test datasets along with their tissue type and the number of nuclei screened by the regression-based ranking with a posterior probability greater than 0.1. This threshold was manually determined by visual examination in order to demonstrate proof-of-concept. While such a threshold seems intuitively low, this is because the distribution of segmentation probabilities was concentrated near zero, an indication that most objects had some attribute penalized by the logistic regression. The NCBD dataset corresponded to a benign breast disease for which the spatial localization of the above genes had a high degree of similarity to that of normal tissue samples. The remainder of the table shows the probability of similarity in gene distribution for both *FRA2* and *HES5* between the pooled normal dataset vs. the manually/automatically screened nuclei. Green text indicates statistical significance.

**Table 3:** Comparison of the automated method with manual analysis shows acceptable agreement

**Dataset**	**Type**	**# Nuclei**	**Manual *FRA2***	**Auto *FRA2***	**Manual *HES5***	**Auto *HES5***	**Agree *FRA2***	**Agree *HES5***
D1	NCBD	63	0.1132	0.1918	0.1363	0.1321	*✓*	*✓*
D2	Cancer	164	0.2689	0.4360	0.0000	0.0000	*✓*	*✓*
D3	Cancer	115	0.0050	0.0001	0.0003	0.0000	*✓*	*✓*
D4	Cancer	133	0.0000	0.0001	0.0000	0.0000	*✓*	*✓*
D5	Cancer	188	0.0237	0.0000	0.0000	0.0000	X	*✓*
D6	Cancer	147	0.0004	0.0182	0.5239	0.5019	X	*✓*
D7	Cancer	63	0.0000	0.0000	0.0724	0.0013	*✓*	X
D8	Cancer	174	0.0002	0.0000	0.3017	0.3576	*✓*	*✓*
D9	Cancer	120	0.0000	0.0000	0.0000	0.0000	*✓*	*✓*
D10	Cancer	117	0.0001	0.0005	0.0157	0.0201	*✓*	*✓*
D11	Cancer	87	0.0001	0.0007	0.0000	0.0286	*✓*	X
D12	Cancer	153	0.0009	0.0048	0.0000	0.0130	*✓*	X
D13	Cancer	37	0.0000	0.0004	0.0526	0.9827	*✓*	*✓*
						Total:	11/13	10/13
							84.60%	76.90%

^a^13 test datasets with their tissue type (NCBD = non-cancerous breast disease) and the number of nuclei screened with a probability above 0.1.

The number of nuclei analyzed was required to be approximately 100 or above for a statistically significant result. A sufficient number of nuclei was screened in most of the datasets, the exceptions being D1, D7 and D13. Manual inspection of these datasets indicated nuclei clustering was causing a poor quality segmentation. Thus, the shortage of usable nuclei was not due to algorithmic insensitivity, but was rather a result of too few quality candidate segmentations. More images should be acquired in such a situation.

The spatial localization statistics for both the genes obtained manually and automatically were compared to that of a pooled normal dataset, which contained the statistics of nine normal samples aggregated together and have been published previously [24]. Comparison was done using the Kolmogorov-Smirnov test with a significance level of *α *= 0.01 (a test sample was considered to be different from the control group if the probability of it not being significantly different was less than 1%). Table 3 shows the probability of similarity in gene distribution for both *FRA2* and *HES5* between the pooled normal dataset vs. the manually/automatically screened nuclei. The automatic results differed from the manual result in only two cases for *FRA2* and 3 cases for *HES5* (red X’s). This demonstrates the efficacy of the automatic method with respect to an entirely manual analysis and compares favorably to previous results reported in [23].

### FISH centrality is stable

**Figure 4:**
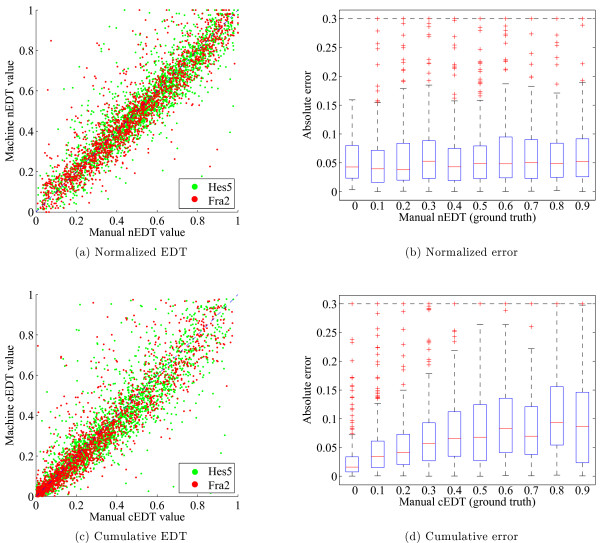
**(a) Scatterplot of the normalized man vs. machine EDT.** This is a comparison of the nEDT vs. cEDT values between fully manual and machine segmentation of 1086 nuclei, comprising 3720 gene markers. **(b)** Normalized EDT error as a function of position within the nucleus (0 = center, 1 edge) **(c)** Scatterplot of the cumulative man vs. machine EDT. **(d)** Cumulative EDT error as a function of position within the nucleus. FISH spots close to the periphery have larger error for cEDT, while those for nEDT are not position dependent. The red line is the median, the box extends to the 25th and 75th percentiles, the whiskers are the most extreme data points not considered outliers, outliers are plotted as red “+”s. Outliers above 0.3 not shown to improve visualization.

Demonstrating stability of gene position measurements in the presence of segmentation noise is a necessary requirement for automation. If the position measurements are highly susceptible to small perturbations in the segmentation contour, then it is difficult to evaluate the effectiveness of the ranked retrieval. To demonstrate agreement, 1086 randomly selected nuclei were manually segmented using a tablet device. The cumulative and normalized gene position measurements were computed for FISH signals for both the manual and automatic segmentations. For the normalized EDT values, the man-machine correlation coefficient was *r *= 0.91(*p *< 0.05) with a mean absolute error of 0.065. For the cumulative EDT values, the man-machine correlation coefficient was *r *= 0.90(*p *< 0.05) with a mean absolute error of 0.070. This agreement was found to give satisfactory results for localization. Scatter plots of both are shown in Figure 4. We refer the reader to the Additional file 1 for further discussion on spatial errors in the cumulative EDT.

## Discussion

### Expert agreement

Part of the variation in the human agreement is attributable to justifiable, subjective opinion differences. Such disagreement forms a theoretical ceiling on the best performance expected from a supervised method. The other portion of the error, that attributable to human mistakes such as fatigue, mood, varying tolerances, or the influence of memory and context, does not affect the computer’s performance.

It is tempting to attribute the low manual agreement to a lack of expertise, namely that a panel of pathologists would show more consensus than less experienced reviewers. While this is certainly a question worthy of further experimentation, we note that the task of identifying segmented DAPI nuclei at high magnification is *not* one that requires particular histological expertise, in addition to being too time consuming to reasonably expect a pathologist to handle. For example, manual annotation of the 43,956 nuclei in this experiment required several months of full-time work per reviewer, even with a dedicated software interface to expedite the job. Results from training on subsets of the three annotations indicated that multiple annotations gave diminishing returns for the effort invested. The best training set came from all three reviewers, but the advantage was modest and did not indicate that multiple reviewers were vital to performance.

It is possible the majority of the observed differences in annotation were the result of variability in qualitative opinions, such as how much of a nucleus was occluded, how much of a nucleus needed to be in the focal plane to be labeled usable, and how much accuracy is necessary for the segmentation boundary. It remains an open question of how these ROC values would change under different reviewers.

### Dataset quality

The quality measure proposed in this manuscript may act as a useful tool when acquiring images at the microscope. Quality was found to correspond to the difficulty of segmentation across different images. Since the regression performs with high specificity, multiplying the dataset quality by the number of candidate objects gives a rough estimate of the nuclei yield. For example, if 200 nuclei are necessary for a given screening application, images can be acquired, segmented and ranked immediately, until the desired number of usable nuclei are captured. For benign epithelial tissue packed into tight glands with touching cells, this may require 40 images. For a disorganized population of scattered tumor cells, it may require only 10 images.

Standard fluorescence confocal images (neglecting the Z-stack) are a 2D slice of three-dimensional structures into two dimensions, meaning the observed radial distributions are not necessarily the true radial distributions. In the most simple approximation, one where the nuclei are modeled as spheres, a 2D projection has the effect of shifting the distribution towards the center (a boundary point may falsely appear at the center, but a center point cannot shift towards the boundary). However, no such general claim can be made for the case of non-spherical shapes. We therefore adopt the assumption that the nuclei do not have a preferred orientation, or equivalently, that the bias introduced by the projection is averaged out over a large-enough sample. One might try to avoid this assumption by analyzing 3D images, however the practical difficulties of sectioning, staining, acquiring, and analyzing such images are currently prohibitive. While it is possible that information in the third dimension may be less useful than rapidly imaging a larger sample size of 2D images [27], this determination depends on the application and must be made on a case-by-case basis.

### Reviewing localization results

The benefit of ranking segmentations according to a probability is apparent when viewed in the context of a high-throughput workflow. Here, the investigator must choose one of four options for studying gene position. In order of decreasing manual burden, they are: fully manual nuclei selection and segmentation, automatic segmentation followed by manual selection, automatic segmentation followed by automatic binary classification, and finally, automatic segmentation followed by ranking. The choice to rank the output provides a natural means to review the segmentations, stepping down the list and manually discarding the worst false positives until a satisfactory sample or cutoff point is reached. Data points farthest from the decision margin are more likely correctly classified, which makes reviewing the ranked output less cumbersome than dealing with an unsorted, binary class label. The probabilistic output may also alleviate the need pick a fixed number of nuclei for analysis, whether by number or threshold. For example, the contribution of an individual FISH marker to the position distribution could be weighted by the object’s posterior probability, thereby discounting the position measurements from incorrectly segmented nuclei. Software using the ranking methodology is currently under evaluation.

Future experimentation is necessary to determine if the nuclei selected by an automatic or manual method are unbiased, meaning they are statistically representative of the cell population under study. It was observed that the automatic method presented in this paper ranked perimeter/area among the most salient attributes of a proper segmentation. This preference was also observed in the nuclei selected by the human reviewer, who tended to choose convex, round nuclei. A larger study is necessary to answer whether there is a reproducible bias towards round nuclei, whether the human and machine are similarly biased, and whether the radial distributions are altered due a biological process affecting the nuclear shape.

It is beyond the scope of this work to describe the specific features which capture the full range of ways in which different segmentation methods can fail. Instead, the methodology is offered as segmentation-agnostic approach to quality assessment. The features most useful for discrimination are, in part, dependent on the segmentation method used in each specific application. For example, a classifier trained to distinguish properly segmented images with the watershed transform is not expected to perform ideally on different images segmented with active contours. However, given a feature space captures the variance between acceptable and unacceptable segmentations, ranked retrieval by logistic regression is a suitable way to exploit this variance and quality check the results.

## Conclusions

While many quality segmentation methods are available to find nuclear borders, these methods do not ensure that the selected population is usable for localization analysis. The heterogeneity in microscopic images makes segmentation assessment necessary to achieve this end. In this paper, a ranked retrieval approach using logistic regression was introduced as a means to perform segmentation assessment. This approach was shown to outperform the inter-observer human agreement on a breast cancer TMA dataset containing over 40,000 candidate nuclei, with a mean ROC area of 0.95 when trained in a leave-one-subject-out fashion. These results indicated gene position could be both automatically and precisely measured. When compared to a completely manual analysis, the automatic results agreed with manual measurement in 21 out of 26 statistical comparisons of cancer tissue and non-cancer breast disease tissue to a pooled normal distribution. Due to its generality, this ranked retrieval method could be applied to other high-throughput applications that rely on accurate segmentation of cells or nuclei.

## Competing interests

The authors declare that they have no competing interests.

## Author’s contributions

WJC was primary author of the manuscript and responsible for the design & implementation of the ranked retrieval methodology. PRG and KN implemented 2D cell nuclei segmentation, aided with algorithm conception, design, & implementation, as well as the analysis & interpretation of data. PRG authored portions of the background and methods. KJM acquired and manually analysed the data, contributed towards design & interpretation of results. TM was responsible for conception & design, interpretation of results. DJF and SJL supervised the experiments and provided analysis and interpretation of data. All authors read and approved the final manuscript.

## Supplementary Material

Additional file 1 Spatial Errors in the Cumulative EDT, a detailed confusion matrix for the human reviewers, and a description of features used in the analysis. Click here for file
