# Supplementary material for: Ranked retrieval of segmented nuclei for objective assessment of cancer gene repositioning
Source: BMC Bioinformatics. 2012 Sep 12;13:232. doi: 10.1186/1471-2105-13-232 (PMC3484015; doi:10.1186/1471-2105-13-232)
Supplement: Additional file 1 — Spatial Errors in the Cumulative EDT, a detailed confusion matrix for the human reviewers, and a description of features used in the analysis. [file 1471-2105-13-232-S1.pdf]

Additional Files

[The following material is to appear in an online supplement to the article]

Spatial Errors in the Cumulative EDT

Unlike the normalized form, the cumulative EDT is fully shape-invariant, allowing direct comparison between nuclei which have very different boundaries, have different sizes, or were imaged under different magnifications. However, such invariance comes at the cost that the cEDT values are not equally susceptible to spatial noise. This can be illustrated through the example of a circular nucleus. Consider a FISH spot at position r in a nucleus of radius R. The normalized EDT is given by,

$$nedt(r) = 1 - \frac{R-r}{R} = \frac{r}{R}. \tag{6}$$

The cumulative EDT is the area ratio between the circles of radius r and R,

$$cedt(r) = (r/R)^2. \tag{7}$$

Consider the effect of a perturbation in the radial direction (as might be introduced by a segmentation error). Letting $r \to r + dr$, the change in normalized EDT is proportional to the added noise,

$$nedt(r + dr) = \frac{r}{R} + \frac{dr}{R}, \tag{8}$$

whereas the effect on the cumulative distribution has a radius-dependent term,

$$cedt(r + dr) = \frac{r^2 + rdr + dr^2}{R^2} \approx \frac{r^2}{R^2} + \frac{r\,dr}{R^2}. \tag{9}$$

Noting that rdr is the “area element” of a cylindrical ring, this shows that errors affect the cumulative distribution function more at the periphery of the object compared to the center. This effect was empirically observed in the data (Figure 4). There are two points to discuss from these results. Firstly, the machine EDT values are systematically biased towards the nuclear center. This is an artifact from the manual segmentation being generally more smooth than the automatic. Secondly, the cEDT values are more noisy closer to the nuclear boundary and less noisy in the center, compared to the nEDT method. In this study, the cEDT was chosen for reasons of shape invariance. However, more comprehensive future work is warranted to determine whether the inhomogeneous spatial noise of the cEDT or the shape-dependence of the nEDT yields the most accurate position measurement.

Table 4: Confusion matrix for three different reviewers (y_1, y_2, y_3) for three classes of segmentation (good, maybe, reject)

	y_2 good	y_2 maybe	y_2 reject
y_1 good	1476	253	471
y_1 maybe	862	454	1551
y_1 reject	478	525	37886
	y_3 good	y_3 maybe	y_3 reject
y_1 good	1118	321	761
y_1 maybe	627	528	1712
y_1 reject	525	946	37418
	y_3 good	y_3 maybe	y_3 reject
y_2 good	1535	514	767
y_2 maybe	247	316	669
y_2 reject	488	965	38455

Features — Description of Features used in the analysis

This section provides the name, range, description, and (when it can be succinctly written) an appropriate formula for features to quantify nuclear segmentation quality. Whenever appropriate, features were normalized by area (if the units are 2-D) or perimeter (if the units are 1-D) in order to achieve basic scale invariance. Since the orientation of the images is random, rotationally asymmetric features were avoided.

A: Segmentation mask area
P: Segmentation mask perimeter
$\mathbf{M}$: 2-D Segmentation mask
$\mathbf{E}$: 2-D Best-fit ellipse mask
$\vec{p}_i \in \mathbf{P}$ Set of pixels belonging to perimeter
$\vec{y}_i \in \mathbf{M}$ Set of pixels inside mask

M Morphological features: relates only to border shape

T Texture features: relates to image contents within the segmentation border

F FISH features: relates to FISH (fluorescent in-situ hybridization) spots

C Contextual features: relates to entire image containing multiple nuclei

Morphological

Area

Name: M_area

Range: $\mathbb{N} \in [1, \infty)$

Formula: A

Description: Number of pixels in segmentation mask

Rationale: Quantifies nucleus size

Perimeter

Name: M_perimeter

Range: $\mathbb{N} \in [1, \infty)$

Formula: P

Description: Length of segmentation boundary

Rationale: Quantifies nucleus border length

Perimeter to area ratio

Name: M_p2a

Range: $\mathbb{R} \in [1, \infty)$

Formula: $P^2/(4\pi A)$

Description: Perimeter to area ratio

Rationale: Quantifies roundness of nucleus, 1 is a perfect circle, higher numbers are more jagged shapes

Solidity

Name: M_solidity

Range: $\mathbb{R} \in (0, 1]$

Formula: A/A_{ch}

Description: Ratio of area to the convex hull area

Rationale: Quantifies area of of border indents

Convex hull perimeter to object perimeter ratio

Name: M_cvhullp2p
Range: $\mathbb{R} \in (0, 1]$
Formula: P/P_{ch}
Description: Ratio of convex hull perimeter to object perimeter
Rationale: Quantifies length of border indents

Maximum inscribed circle ratio

Name: M_inscribedcircle2a
Range: $\mathbb{R} \in [0, 1]$
Formula: A_{circle}/A
Description: Ratio of the area of the largest circle which can be inscribed in the boundary to the total area
Rationale: Quantifies deviation of boundary from a circle

Ellipse eccentricity

Name: M_eccentricity
Range: $\mathbb{R} \in [0, 1]$
Formula:
Description: Eccentricity of the ellipse that has the same second-moments as the mask
Rationale: Quantifies amount to which the nucleus shape is an ellipse vs. a circle

Best-fit ellipse error area ratio

Name: M_irregularitya
Range: $\mathbb{R} \in [0, \infty]$
Formula: $(\mathbf{M} \oplus \mathbf{E})/A$
Description: Area difference between best-fit ellipse and boundary
Rationale: Quantifies amount the border differs from an ellipse

Length

Name: M_length
Range: $\mathbb{R} \in [\,0,\infty)$
Formula: $\frac{P}{4} + \sqrt{\frac{P2}{16} - A}$
Description: Length approximation
Rationale: Quantifies amount of elongation

Width

Name: M_width
Range: $\mathbb{R} \in [\,0,\infty)$
Formula: $\frac{P}{2} - Length$
Description: Width approximation
Rationale: Quantifies amount of width

Mean pairwise distance between perimeter points

Name: M_meandist
Range: $\mathbb{R} \in [\,0,\infty)$
Formula: $\frac{1}{|\mathbf{P}|} \sum_i \sum_{j \neq i} d(\vec{p}_i, \vec{p}_j)$
Description: Mean all-pairs distance between points on perimeter
Rationale: Correlates with size, but is slightly shape-dependent

Standard deviation of polar histogram

Name: M_stdang
Range: $\mathbb{R} \in [\,0,\infty)$
Formula:
Description: Shift the perimeter coordinates so the origin is at the center of mass. Transform the perimeter points to polar coordinates and construct a histogram of the angular component. This is the standard deviation of that histogram.
Rationale: Measures "isotropy" in the amount of border points at a given angle from the center

Number of severe corner points

Name: M_numcornerp
Range: $\mathbb{R} \in [0, \infty]$
Formula: $N_{corners}/P$
Description: Uses a minimum eigenvalue corner detector along with a threshold to suppress weak corners, normalized by the perimeter length so that larger nuclei are not penalized
Rationale: Nuclei should have fewer strong corners

Box-counting dimension (Fractal dimension)

Name: M_boxcountdim
Range: $\mathbb{R} \in [0, \infty)$
Formula: $\lim_{\epsilon \to 0} -\frac{\log N(\epsilon)}{\log(\epsilon)}$, where ϵ is the box size and $N(\epsilon)$ is the number of boxes required to cover the set
Description: Measure of the fractal dimension of the perimeter. The number of boxes necessary to cover the boundary are determined at finer and finer scales. The number of boxes is plotted on a log scale with the box size. The negative of the slope is the box-counting dimension.
Rationale: Quantifies complexity of segmentation boundary

Erosion Profile

Name: M_halfscore, M_maxnumcomps
Range: $\mathbb{R} \in [0, 0.5)$, $\mathbb{N} \in [1, \infty)$
Formula: $\max_i \left(\frac{d}{di}(A_i/A_{i-1}) \right)$
Description: Erode the mask repeatedly with a one-pixel disk until it vanishes. At each step, record the number of connected components as well as the ratio of the current largest connected component to the area of the largest connected component at the previous step. The (discrete) derivative of this ratio approaches 0.5 when the mask breaks into two connected components with approximately equal size. This is called the "half score."
Rationale: The half score looks to identify segmentations with narrow passages separating large areas. When the narrow passage is eroded away, the area fraction jumps in a discontinuous manner. Smooth segmentations of nuclei should also not break into many connected components as they are eroded.

Minimum elliptical Fourier coefficients

Name: M_numFourierCoeff
Range: $\mathbb{N} \in [1, 100]$
Formula: $\min_i [mask \oplus fourier\_mask(i)/A] < 0.10$
Description: Number of elliptical Fourier coefficients necessary to reconstruct a mask to within 10% area error of the segmentation mask. We cap the number at 100 for practical purposes.
Rationale: Real nuclei are mostly simple/elliptical and should require fewer Fourier coefficients to accurately reconstruct

Texture

Mean intensity

Name: T_meanintensity
Range: $\mathbb{R} \in [0, 1]$
Formula: $\frac{1}{|\mathbf{M}|} \sum_{i \in \mathbf{M}} y_i$
Description: Mean of grayscale intensity inside nucleus
Rationale: Quantifies amount of DAPI staining, nuclear material

Intensity range

Name: T_intensityrange
Range: $\mathbb{R} \in [0, 1]$
Formula: $\max_i(y_i) - \min_i(y_i)$
Description: Range of grayscale intensity inside nucleus
Rationale: Quantifies global variation in DAPI staining, should be smaller for correct segmentations (which do not contain background)

Entropy

Name: T_entropy
Range: $\mathbb{R} \in [0, \infty)$
Formula: $-\sum(h \log_2(h))$, where h are image histogram counts
Description: Global entropy of grayscale values

Rationale: Quantifies global variation in nuclear texture

Gray-level co-occurrence matrix derived statistics

Name: T_contrast, T_correlation, T_energy, T_homogeneity

Range: $\mathbb{R} \in [0, (size(GLCM) - 1)^2]$, $\mathbb{R} \in [-1, 1]$, $\mathbb{R} \in [0, 1]$, $\mathbb{R} \in [0, 1]$

Formula: $\sum_{i,j} |i - j|^2 g_{ij}$, $\sum_{i,j} \frac{(i-\mu i)(j-\mu j)g_{ij}}{\sigma_i \sigma_j}$,$\sum_{i,j} g_{ij}^2$, $\sum_{i,j} \frac{g_{ij}}{1+|i-k|}$

Description: Statistics describing the gray-level co-occurrence matrix

Rationale: Quantifies general properties of texture: contrast is 0 for a constant image, correlation is 1 or -1 for a perfectly positively or negatively correlated image, correlation is NaN for a constant image, energy is 1 for a constant image, homogeneity is 1 for a diagonal GLCM

FISH

Multiple FISH species can inhabit the same slide, so each feature can be applied to each FISH channel separately. A 'r' or 'g' is added to distinguish the red and green FISH signals in the feature extraction here.

Number of FISH spots

Name: F_g, F_r

Range: $\mathbb{N}$

Formula:

Description: Number of FISH spots

Rationale: FISH only binds to nuclear material, is necessary for analysis

FISH per area

Name: F_g2a, F_r2a

Range: $\mathbb{R}$

Formula: N_{FISH}/A

Description: Number of FISH spots normalized by area

Rationale: More equal comparison of "amount" of FISH than the absolute count

FISH convex hull area

Name: F_gch2a, F_rch2a, F_grch2a
Range: $\mathbb{R} \in [0, 1]$
Formula: A_{ch}/A
Description: Ratio of convex hull area formed on FISH spots to total area
Rationale: Quantifies the extent to which the FISH spots span the nucleus

FISH convex hull boundary crossing

Name: F_gchbound, F_rchbound
Range: 0 or 1
Formula:
Description: Does the convex hull formed by the FISH spots intersect the nuclear boundary?
Rationale: Possible marker for badly segmented objects

Mean distance between FISH spots

Name: F_gmean, F_rmean
Range: $\mathbb{R}$, NaN
Formula:
Description: Mean distance between FISH spots of a given species, is NaN when there are 1 or 0 FISH markers
Rationale: Quantifies the separation of FISH spots
